# The Expression of CNS-Specific *PPARGC1A* Transcripts Is Regulated by Hypoxia and a Variable GT Repeat Polymorphism

**DOI:** 10.1007/s12035-019-01731-5

**Published:** 2019-08-30

**Authors:** Selma M. Soyal, Petra Bonova, Markus Kwik, Greta Zara, Simon Auer, Cornelia Scharler, Dirk Strunk, Charity Nofziger, Markus Paulmichl, Wolfgang Patsch

**Affiliations:** 1 Institute of Pharmacology and Toxicology, Paracelsus Medical University, 5020 Salzburg, Austria; 2 Institute of Neurobiology, Biomedical Research Center of the Slovak Academy of Sciences, Bratislava, Slovak Republic; 3 Institute for Medical and Chemical Laboratory Diagnostics, Paracelsus Medical University, 5020 Salzburg, Austria; 4 Institute of Experimental and Clinical Cell Therapy, Spinal Cord Injury and Tissue Regeneration Center, Paracelsus Medical University, 5020 Salzburg, Austria; 5 PharmGenetix GmbH, Niederalm, 5081 Salzburg, Austria; 6 Department of Personalized Medicine, Humanomed, 9020 Klagenfurt, Austria

**Keywords:** *PPARGC1A*, PGC-1α, CNS-specific isoforms, FOXA2, ESRRA, Hypoxia, Microsatellite

## Abstract

*PPARGC1A* encodes a transcriptional co-activator also termed peroxisome proliferator-activated receptor (PPAR) gamma coactivator 1-alpha (PGC-1α) which orchestrates multiple transcriptional programs. We have recently identified CNS-specific transcripts that are initiated far upstream of the reference gene (RG) promoter. The regulation of these isoforms may be relevant, as experimental and genetic studies implicated the *PPARGC1A* locus in neurodegenerative diseases. We therefore studied cis- and trans-regulatory elements activating the CNS promoter in comparison to the RG promoter in human neuronal cell lines. A naturally occurring variable guanidine thymidine (GT) repeat polymorphism within a microsatellite region in the proximal CNS promoter increases promoter activity in neuronal cell lines. Both the RG and the CNS promoters are activated by ESRRA, and the PGC-1α isoforms co-activate ESRRA on their own promoters suggesting an autoregulatory feedback loop. The proximal CNS, but not the RG, promoter is induced by FOXA2 and co-activated by PGC-1α resulting in robust activation. Furthermore, the CNS, but not the RG, promoter is targeted by the canonical hypoxia response involving HIF1A. Importantly, the transactivation by HIF1A is modulated by the size of the GT polymorphism. Increased expression of CNS-specific transcripts in response to hypoxia was observed in an established rat model, while RG transcripts encoding the full-length reference protein were not increased. These results suggest a role of the CNS region of the *PPARGC1A* locus in ischemia and warrant further studies in humans as the activity of the CNS promoter as well as its induction by hypoxia is subject to inter-individual variability due to the GT polymorphism.

**Electronic supplementary material:**

The online version of this article (10.1007/s12035-019-01731-5) contains supplementary material, which is available to authorized users.

## Introduction

Peroxisome proliferator–activated receptor gamma co-activator 1A (PGC-1α) encoded by *PPARGC1*A, is a versatile transcriptional co-activator involved in multiple transcriptional programs [1–3]. A key issue to understand the diverse functions of PGC-1α is to gain insight into the mechanisms that confer specificity to its interactions with numerous transcription factors. The regulation of PGC-1α function occurs at several levels. Expression of PGC-1α and the transcription factors co-activated by it are critical for some pathways [4]. Furthermore, PGC-1α is targeted by several signaling pathways at the posttranslational level, as site-specific phosphorylation [5, 6], acetylation [7], ubiquitination [8, 9], and methylation [10] have been shown to alter the stability and/or activity of PGC-1α. Alternative splicing and/or transcriptional initiation represent another level of control that results in gain or loss of domains interacting with signaling targets. The truncated NT-PGC-1α resulting from alternative splicing contains the N-terminal activation and nuclear interaction domains and displays functional differences in comparison to the full-length protein [11, 12]. In skeletal muscle, two novel promoters have been described 14 kbp upstream of the reference promoter that are differentially regulated in response to metabolic cues such as fasting and exercise [13, 14]. In the human liver, an alternative promoter in intron 2 of the reference gene gives rise to a 75-kDa protein that showed subtle differences in co-activation selectivity compared to the reference protein [15]. We recently described several distinct CNS-specific *PPARGC1A* mRNAs that are transcribed from a novel promoter located ~ 586 kbp upstream of the reference promoter. These transcripts are more abundant than reference gene (RG) mRNAs in human post-mortem brain samples and are partially conserved in rodents [16].

PGC-1α activates transcriptional programs that are relevant for neurodegenerative diseases such as mitochondrial biogenesis and function, the defense against reactive oxygen species, and autophagy [17–21]. Indeed, several functional studies in animal or cell culture models suggest that reduced PGC-1α function plays a role in neurodegenerative diseases [22–27]. In addition, PGC-1α has been implicated in multiple sclerosis [28, 29]. Genetic studies in humans have suggested associations of the *PPARGC1A* locus with Alzheimer’s, Huntington’s, and Parkinson’s disease and amyotrophic lateral sclerosis [16, 30–36]. As some of these associations involved the CNS-specific region of the *PPARGC1A* gene, it is reasonable to suspect that the CNS-specific isoforms and/or their regulation play a role in these disorders. While the signaling pathways that converge at the RG promoter have been identified in mouse muscle and liver [reviewed in 37] as well as in human tissues and cell lines [15, 38], virtually nothing is known about the regulation of the CNS-specific promoter with the exception of its activation by USF1 [16]. We therefore have compared the regulation of the CNS-specific and RG *PPARGC1A* promoters in human neuronal cell lines and a rat model of ischemia. We identified not only similarities but also substantial differences in the transcriptional regulation of the two promoters.

## Materials and Methods

### Plasmids

Expression plasmids pPGC-1α [39], B4-PGC-1α, B5-PGC-1α, B4-7a-PGC-1α, B5-7a PGC-1α [16], and FOXA2 [39] were described previously. The human ESRRA (NM_001282450) ORF Clone in pcDNA 3.1^+^/C-(K)-DYK was purchased from GenScript (Piscataway, NJ, USA). HA-HIF-1α-pcDNA3 and HA-HIF-2α-pcDNA3 were gifts from William Kaelin (Addgene plasmids #18949 and #18950 respectively [40]). Long and short promoters of both the RG [15] and CNS-specific *PPARGC1A* [16] have been described. Short CNS-specific promoters with increasing guanidine thymidine (GT) sizes (602 bp, − 539 to + 63 relative to the transcription start site in promoters containing 11 GT) were cloned into pGL4.11[*luc2P*] (Promega, Madison, WI, USA) from DNA of subjects that harbored identical CNS promoter sequences but differed in their GT repeat size. A very short promoter luciferase construct (− 84 to + 63 bp relative to the transcription start site) was produced by cloning amplification products of the proximal promoter into pGL4.11[*luc2P*] (Promega). Primers used are shown in Supplementary Material, Table S1.

### Cell Culture and Transfection Experiments

SH-SY5Y and NTERA-2cl.D1 (NT2/D1) cells were obtained from ATCC and cultured in DME (D6046)/F12 1:1 (Sigma-Aldrich, St. Louis, MO, USA) and DME (D0547)/F12 (Sigma-Aldrich) supplemented with 0.21% NaHCO_3_, respectively. The murine hippocampal cell line HT22 was a kind gift from D. Schubert, Salk Institute, La Jolla, CA, USA and cultured in DME (D0547)/F12 1:1 supplemented with 0.21% NaHCO3. Media for all cells were supplemented with 10% fetal bovine serum and 1% penicillin/streptomycin (Invitrogen, Carlsbad, CA, USA). All cells were cultured in a humidified 5% CO_2_ atmosphere at 37 °C. Hypoxia culture experiments were performed in an O_2_-regulatable incubator (Binder GmbH, Tuttlingen, Germany) or in a H135 HEPA hypoxystation (Don Whitley Scientific Limited, West Yorkshire, UK) in a humidified 5% CO_2_ and 1% O_2_ atmosphere. Ciclopirox olamine and deferoxamine were obtained from Sigma-Aldrich. Cells were plated in 24-well plates 1 day before transient transfection with plasmid constructs for 24–48 h using Lipofectamine 3000 (Invitrogen) or DNAfectinTM Plus (ABM Inc., Vancouver, Canada). The pRL-TK plasmid (Promega) was used as a transfection control. Luciferase activities were measured using the Dual Luciferase Reporter Assay System (Promega) as described [15]. Results are representative of two or more experiments, each performed in quadruplicate, and are shown as means + SD.

### RNA and Protein Extraction

Total RNA from SH-SY5Y, NT2/D1 cells, and rat brain regions was extracted using the QIAzol Lysis Reagent (Qiagen, Hilden, Germany) and the RNeasy Lipid Tissue Mini kit (Qiagen). Concentrations of RNA were measured using the Nanovue Plus spectrophotometer (GE Healthcare, Chicago, IL, USA). In addition, the QIAxcel Advanced Instrument (Qiagen) was used to ascertain RNA quality. RNA integrity scores were between 8 and 9 for all samples used. For total protein preparation of whole cultured cells, cell pellets were solubilized in RIPA buffer containing protease inhibitors (HaltTM Protease Inhibitor Cocktail, Thermo Fisher Scientific, Waltham, MA, USA). Clear supernatants were obtained after centrifugation (10,000×*g* for 20 min at 4 °C) of suspensions that were intermittently incubated on ice and heated at 95 °C and sonicated (Sonoplus HB 27, Bandelin, Berlin, Germany). Nuclear and cytoplasmic extracts were prepared using the NE-PER Nuclear and Cytoplasmic Extraction Reagents Kit (Thermo Fisher Scientific) according to the manufacturer’s protocol. Protein concentrations were determined by the RCDC Protein Quantification Assay (Bio-Rad Laboratories, Inc. Hercules, CA, USA).

### Western Blot Analysis

Immunoblotting was performed as described [16]. In brief, equal amounts of NT2/D1 cell extracts (20 or 40 μg) were subjected to SDS electrophoresis in 7–10% polyacrylamide gels. Separated proteins were transferred to polyvinylidene fluoride membranes (Bio-Rad Laboratories, Inc.) that were blocked with Tris-buffered saline (TBS), pH 7.6, containing 10% Blotting Grade Blocker (Bio-Rad Laboratories, Inc.) for 2 h at room temperature. Membranes were incubated with the primary antibodies directed against HIF1A (HIF-1α, NB100-105, Novus Biologicals, Centennial, CO, USA), diluted 1:1000 in 0.1% Tween 20 in 1× TBS overnight at 4 °C. Membranes were then incubated for 1 h at room temperature with the respective secondary antibody (IRDye® 800CW Goat anti-Rabbit IgG(H + L) or IRDye® 800CW Goat anti-Mouse IgG(H + L) from LI-COR, Lincoln, NE, USA) and analyzed using the ODYSSEY infrared imager (LI-COR). β-Actin (#4967, Cell Signaling technology, Danvers, MA, USA) or Histone H3 (NB500-171, Novus Biologicals) antibodies were used as loading controls. For densitometric analysis of Western blots, Image Studio™ software (LI-COR) was used.

### Real Time PCR

DNase I-treated total RNA from cell cultures (1 μg/reaction) was reverse transcribed with the QuantiTect Reverse Transcription kit (Qiagen), using a mix of random hexamers and oligo-dT primers. cDNAs were amplified in duplicate by real-time PCR using Maxima SYBR Green (Thermo Scientific) or GoTaq qPCR Master Mix (Promega) and primers targeting exons B1 and B4 or B5 and exon 2 to quantify the two main CNS-specific *PPARGC1*A transcripts. Primers targeting exon 1 and exon 2 were used to target RG transcripts. The transcripts encoding the class of NT-PGC-1α isoforms were quantified by targeting exon 5 and exon 6A (Supplementary Material, Table S1 for primer sequences). To directly compare the amounts of *PPARGC1A* transcripts, gene segments containing the sequences targeted by the respective transcript-specific assays were cloned and used for the construction of standard curves to normalize for the efficiency of assays. Primers to quantify *VEGF* transcripts are also shown in Table S1. Total RNA from rat brain regions was reverse transcribed as described above. As the B5 encoded isoform is not present in rats, we measured *B1b4*, exon 1, exon 2, exon 5, and exon 6A containing transcripts as well as transcripts encoding VEGF and GLUT1. The respective primers are shown in Supplementary Table S1. For transcript amplification, the Rotor-Gene TM Q (Qiagen) instrument or the LightCycler TM 480 Instrument (Roche, Basel, Switzerland) was used. Relative mRNA levels were calculated using the comparative threshold cycle method (Δ_Cr_). Constitutively expressed *RPLP0* (Ribosomal Protein, large, P0) mRNA was used for normalization of mRNA abundance.

### ChIP Assays

The chromatin immunoprecipitation (ChIP)-IT Express Enzymatic kit (Active Motif Europe, La Hulpe, Belgium) was used. Cross-linking of 10^6^ NT2/D1 cells, enzymatic shearing and preparation of input DNA were performed as described [41]. After an overnight incubation with 4 μg of HIF1A-specific monoclonal antibodies at 4 °C, chromatin/DNA complexes were immunoprecipitated using protein G magnetic beads. After DNA purification, primers described in Supplementary Material, Table S1 were used to amplify fragments of the CNS-specific promoter. As positive control, we designed the human equivalent primers of those described [42] spanning an HRE in the mouse *Ntrk2* (*TrkB*) promoter known to be activated by HIF1A. As a negative control, primers spanning Exon *B5* of CNS-*PPARGC1A* that did not contain a predicted HRE were used. IgG instead of the HIF1A antibody was used to confirm the absence of non-specific bands.

### Animal Experiments

The experiments were carried out in accordance with the protocol for animal care approved by European Communities Council Directive (2010/63/EU) with permission of The State Veterinary and Food Administration of the Slovak Republic (4451/14-221 and 4247/15-221) under the supervision of Ethical Council of the Institute of Neurobiology, Bratislava Slovak Academy of Sciences. Every effort was made to minimize animal suffering and reduce the number of animals used. Adult male albino Wistar rats weighing 300–500 g obtained from Velaz (Czech Republic) were housed in the certified vivarium of the Institute of Neurobiology, *Biomedical Research Center* of the Slovak Academy of Sciences, maintained on a 12-h light/dark cycle and given food and water ad libitum. Food was withdrawn 1 day before surgery.

#### Experimental design

Four groups of animals were used: two ischemic groups of 3 rats each with different post-ischemic reperfusion intervals (ischemia 5 min followed by 1 h or 3 h of reperfusion) and two sham control animal groups (*n* = 3 each) sacrificed at the time points of the experimental ischemic animals.

#### Model of ischemia

Transient forebrain global ischemia was induced [43] with modifications [44]. The rats were anesthetized with 4% halothane in anesthetic cages and maintained with 1.5% halothane during surgery. On the first day, both vertebral arteries were irreversibly occluded by coagulation through the alar foramina. The next day, rats were re-anesthetized and the common carotid arteries were dissected free and clamped just before awakening by small atraumatic clips to induce forebrain ischemia. After 5 min of ischemia, blood flow was restored by releasing the clips and verified visually. Normothermic conditions (approximately 37 °C) were maintained by a feedback-controlled heating lamp and pad during all surgical procedures. Animals that became unresponsive within 30 s after clip tightening, lost the righting reflex during bilateral carotid artery occlusion, and showed no seizures during and after ischemia were used for the experiments. Sham-operated controls were surgically treated the same as the ischemic group, but the common carotid arteries were not clamped on the second day.

#### Tissue sample collection

Animals were sacrificed by decapitation under halothane anesthesia; the brains were quickly removed and maintained at 0 °C. The fronto-parietal and occipital cortex, striatum (dorsolateral part and rest of the striatum, respectively), hippocampus, thalamus, and cerebellum were separated. To compare the selectively vulnerable CA1 versus the relatively resistant dentate gyrus and CA3 regions, the hippocampus was divided into the CA1 region and the rest of the hippocampus (DG) under a dissecting microscope. Collected tissue was weighed, divided into halves, and frozen at − 20 °C in 5 volumes of RNAlater (Sigma-Aldrich) until RNA analysis.

### Statistical Analysis

In transient transfection studies, ANOVA was used for comparisons of means and the Tukey honest significance test for post-hoc comparisons. Two-way ANOVA was used to ascertain effects of transcriptional activators, co-activators, and their interactions. ANOVA was used to compare effects of reduced oxygen tension and CPX. Three-way ANOVA was used to analyze effects on transcript levels in the in vivo model of transient ischemia. Treatment (sham/transient ischemia), post-ischemic reperfusion time (1 h/3 h) and brain regions sampled (1–7) were used as independent variables. Reported *p* values are 2-tailed.

## Results

**Fig. 1:**
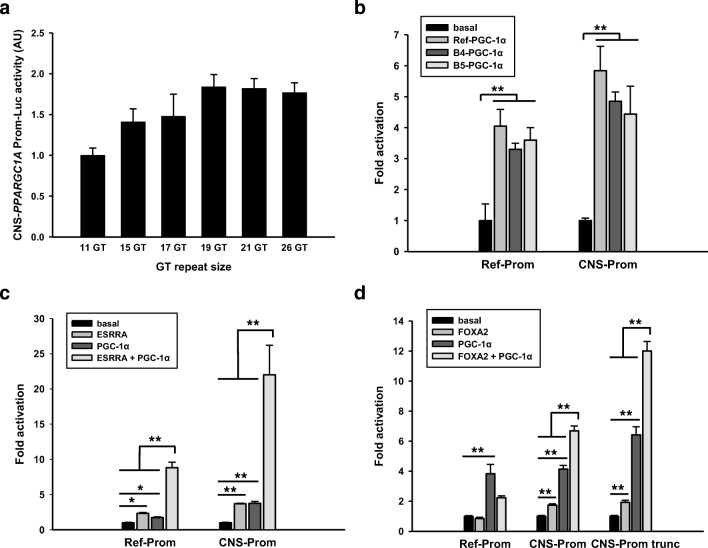
Distinct and common regulations of the reference and the CNS-*PPARGC1A* promoters. A common GT repeat polymorphism in the CNS promoter increases its activity up to a repeat size of 21 GT (*p* < 0.001); proximal CNS promoters from human subjects differing in GT size, but otherwise of identical sequence (− 539 to + 63 bp relative to the transcription start site in the common allele harboring 11 GTs) were cloned in reporter vectors and transiently transfected into SH-SY5Y cells (**a**). Both the reference and the CNS promoters are activated by the reference protein and the brain-specific isoforms B4- and B5-PGC-1α via co-activation of transcription factors not further identified. Equimolar amounts of reporter constructs encoding the reference promoter (2.6 kbp) and the CNS promoter (2 kbp) were transfected into SH-SY5Y cells along with expression plasmids encoding the reference protein or CNS-specific isoforms (**b**). ESRRA activates and PGC-1α co-activates ESRRA on both the reference and the CNS promoter. The reference or CNS promoter reporter constructs were co-transfected with empty expression plasmid or plasmids encoding ESRRA, PGC-1α, or both; *p* < 0.001 for interactions of ESRRA and PGC-1α on both the reference and the CNS promoters (**c**). FOXA2 induces the 2.0-kbp CNS, but not the 2.6-kbp reference, promoter and PGC1α co-activates FOXA2 at the CNS promoter. Activation by FOXA2 and co-activation of FOXA2 are maintained on a truncated CNS promoter (− 0.539 to + 63 bp); *p* < 0.001 for interactions of FOXA2 and PGC1α on both the 2.0 kbp and truncated CNS promoters. Similar effects in C and D were observed when reference PGC-1α was substituted by B4-PGC-1α. *p < 0.01; **p < 0.001

We previously identified two microsatellite regions located at − 164 and − 24 bp relative to the transcription start site in the CNS-specific promoter region. The more proximal (GT)_4_GC(GT)_2_GC(GT)_4_G(GT)_3_(GC)_3_ microsatellite showed no variation in DNA from 50 subjects, but the more distal (GT)GC(GT)GC(GT)_N_T(GT)_3_(GC)_5_ microsatellite revealed considerable variation in the number of GT repeats (N). In DNA from 2000 subjects, the GT insertion polymorphism (N) ranged from 9 to 28 repeats with 11 GTs as the most common allele [16]. Both SH-SY5Y and NT2/D1 cells contained 17 GTs. Hence, the numbers indicating the relation to the transcription start site in various clones are given for the 11 GT size, unless otherwise indicated. The instable microsatellite region includes interspersed GC repeats. This region has a high potential for Z-DNA formation [45] known to frequently influence promoter activation [46–48]. To determine possible effects of the variable microsatellite on promoter activity, we amplified proximal CNS promoters (− 539 to + 63 bp relative to the transcription start site) differing in GT sizes, but otherwise containing identical sequences, from human subjects, cloned the fragments into reporter vectors, and transiently transfected the constructs into SH-SY5Y cells. Constructs with increasing GT size displayed increased promoter activity up to 19–21 GTs without further increases by the longest GT inserts (> 21 GTs). Similar, but even more pronounced effects of the 21 GT constructs relative to the 11 GT constructs were observed in NT2/D1 cells and HT22 cells (Fig. 1a; Supplementary Material Fig. S1A, B).

Robust expression of PGC-1α isoforms may be maintained by positive feedback loops. As demonstrated in mouse skeletal muscle, Mef2c binds and activates the reference promoter and co-activation of Mefc*2* by PGC-1α further increases promoter activity resulting in sustained PGC-1α expression [49]. To determine whether the CNS-specific promoter is subject to autoregulation in a human neuronal cell model, SH-SY5Y cells were transiently co-transfected with expression plasmids encoding full-length (FL) reference or CNS-specific proteins and reporter constructs harboring the sequences of the reference (2.6 kbp) or CNS-specific (2 kbp) promoters cloned upstream of the luciferase gene. All promoter constructs were comparably activated by FL reference and the FL-B4 and FL-B5-PGC-1α proteins, even though minor differences in the extent of co-activation were apparent (Fig. 1b). Thus, reference and CNS-specific promoters are co- and cross-regulated by their encoded proteins via co-activation of transcription factors not further identified in these experiments.

We next compared the role of ESRRA in the transcriptional regulation of the reference and CNS-specific promoters. SH-SY5Y cells were transiently co-transfected with reporter constructs harboring the reference promoter (2.6 kbp) and the CNS-specific promoter (2 kbp) along with plasmids expressing ESRRA and FL reference PGC-1α or FL-B4-PGC-1α. Both promoters were significantly induced by ESRRA, the reference FL-PGC-1α, or FL-B4-PGC-1α. A strong synergistic effect of ESRRA and PGC-1α at both promoters was observed (Fig. 1c).

FOXA2, a member of the forkhead family of winged-helix transcription factors, plays an essential role in the development and maintenance of mid-brain neuronal function [50, 51] and has been shown to influence Ppargc1a expression in mice [52]. We therefore compared the transactivation of the reference and CNS promoter by FOXA2 in SH-SY5Y cells in transient co-transfection assays. The CNS-specific promoter, but not the reference promoter, was induced by FOXA2, and synergistic activation of the CNS-specific promoter by FOXA2 and FL-PGC-1α was noted (*p* < 0.001). As we identified putative FOXA2 binding sites in the distal (− 1175 bp) and proximal (− 218 bp) CNS promoter using the PROMO 3.0 transcription factor binding site tool/ALGGEN Research Software (http://alggen.lsi.upc.edu, [53, 54]), we repeated the experiment with a truncated promoter construct comprising − 539 to + 63 bp relative to the transcription start site. As the activation pattern was comparable between the full and truncated CNS-specific promoters, the putative FOXA2 binding site located at (− 218 bp) was likely the cis-functional site (Fig. 1d).

**Fig. 2:**
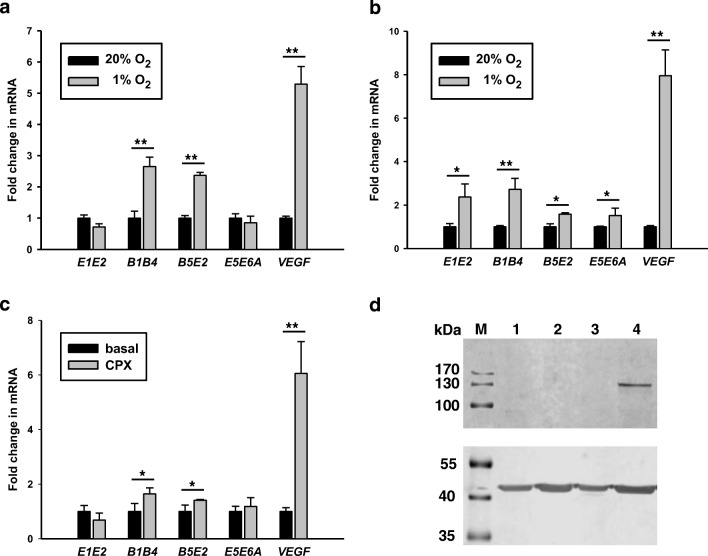
Hypoxia and the iron chelator ciclopirox olamine (CPX) increase the transcript levels of CNS-specific transcripts *B1B4* and *B5E2* and the expression of HIF1A. NT2/D1 cultured in a 1% O_2_ atmosphere for 6 h showed increases in *VEGF*, *B1B4*, and *B5E2* transcripts compared to control cells cultured at 20% O_2_ (**a**). After 24 h of culture, *E1E2* and *E5E6A* transcript levels were also increased (**b**). Incubation of cells with CPX (15 μM) for 24 h resulted in increased levels of *VEGF* and CNS-specific transcripts (**c**). Incubation of NT2/D1 cells with CPX for 24 h increased the expression of HIF1A in the nuclear fraction (top panel); protein extracts from cells incubated without (lanes 1 and 2) or with CPX (lanes 3 and 4); 1 and 3, cytoplasmic fractions; 2 and 4, nuclear fractions; equal amounts of protein (20 μg) were loaded per lane; β-actin shown in the lower panel was used as control; M, molecular weight marker (**d**). *p < 0.01; **p < 0.001

**Fig. 3:**
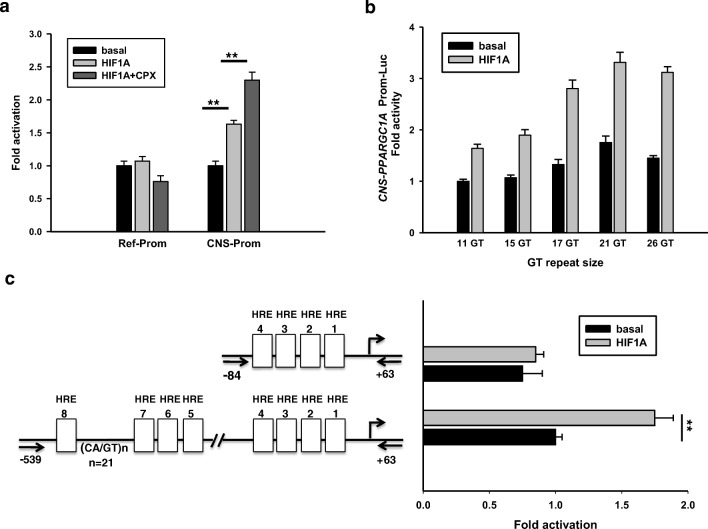
HIF1A activates the CNS, but not the reference, promoter. Reporter constructs containing the reference (2.6 kbp) or CNS promoters (2 kbp, 17 GT repeats) were transfected into SH-SY5Y cells along with expression plasmids encoding HIF1A. Activation of the CNS promoter by HIF1A was further increased by incubation of cells with 15 μmol CPX (**a**). CNS promoter sequences (− 539 to + 63, relative to transcription start as defined by 11 GT repeat size) differing in GT repeat size were cloned in reporter vectors and transfected along with expression vectors devoid of or with insert encoding HIF1A into SH-SY5Y cells. Activities were analyzed by two-way ANOVA and showed significant effects of GT repeat size, HIF1A, and interaction between GT repeat size and HIF1A (all *p* < 0.001) (**b**). Activation of the CNS promoter by HIF1A was not maintained by truncation of the CNS promoter (− 539 to + 63, 17 GT repeats, 8 putative HREs) to − 84 (containing 4 putative HREs), indicating that one or more HREs 5 to 8 are the functional site(s) (**c**)

**Fig. 4:**
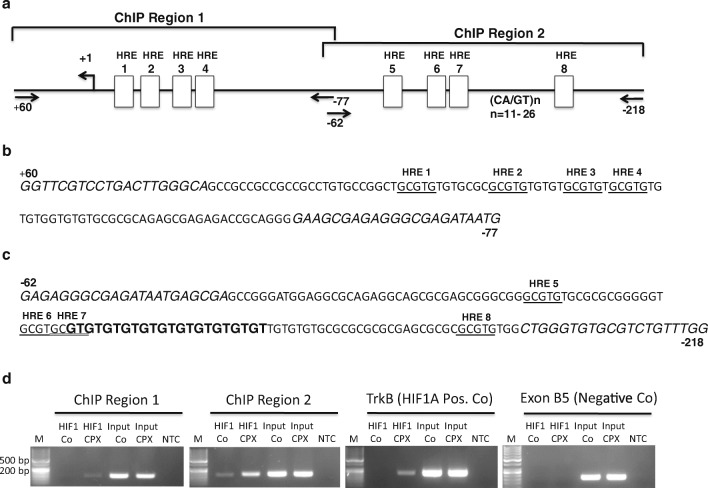
ChIP assays demonstrate binding of HIF1A to a region between − 77 and − 218 relative to the transcription start site and harboring the putative HREs 5 to 8 as well as the GT repeat tract. Schematic describing the location of putative HREs, the GT repeat tract, and primers used to amplify ChIP regions 1 and 2 (**a**). Sequence of ChIP region 1 and 2 with primers (in italics) and putative HREs (underlined); numbers indicate position relative to transcription start site (**b**, **c**). Representative ChIP assays showing interaction of HIF1A with region 2 of the CNS promoter. NT2/D1 cells were cultured for 24 h without or with 15 μmol CPX prior to harvesting of cells. Note that amplification products reflecting the binding of HIF1A were obtained only in NT2/D1 cells treated with CPX. The *TRKB* promoter shown to have a bona fide HIF1A binding site was used as positive control and the CNS-specific exon B5 was used as negative control. Input, 1% of input DNA; NTC no template control

Since neuronal cells are particularly sensitive to ischemia, we studied potential effects of reduced oxygen tension on the expression levels of CNS and RG transcripts in NT2/D1 cells cultured in 20% and 1% O_2_, respectively. After 6 h in culture, levels of the CNS-specific transcripts *B1B4* and *B5E2* as well as *VEGF* transcripts were increased in cultures exposed to 1% oxygen in comparison to cells cultured in 20% O_2_. A similar pattern was noted at 24 h of culture, but average levels of *E1E2* and *E5E6A* transcripts tended also to be higher under oxygen deprived culture conditions (Fig. 2a, b). To determine whether non-hypoxic stimuli, known to stabilize HIF1A via inhibition of proline hydroxylase domain (PHD) isoforms [55, 56], also influenced the expression of *PPARGC1A* transcripts, we incubated cells with the iron chelators ciclopirox olamine (CPX) and deferoxamine (DFO). In NT2/D1 cells, cellular levels of *VEGF* transcripts and CNS-specific transcripts *B1B4* and *B5E2* were increased by CPX, but levels of RG transcripts *E1E2* and *E5E6A* transcripts, encoding NT-PGC-1α, were similar to levels in control cells (Fig. 3c). CPX treatment resulted in clearly detectable levels of HIF1A in cell nuclei as expected (Fig. 3d). In SH-SY5Y cells incubated with DFO, we observed upregulation of *B1B4*, but not *E1E2* transcripts (Supplementary Material Fig. S2). As the latter results were consistent with stabilization of HIF1A and a role of the canonical hypoxia response in the upregulation CNS-specific transcripts, we performed transient transfection studies of SH-SY5Y cells using reporter constructs harboring the CNS-specific and the reference promoter and HIF1A expression plasmids. HIF1A activated the CNS promoter nearly 2-fold, and activation was further increased in the presence of the HIF stabilizer CPX. In contrast, no activation of the reference promoter was observed (Fig. 3a). Plasmids encoding FL reference and/or CNS-PGC-1α activated the CNS promoter as described above, but co-transfection of plasmids encoding HIF1A and FL-PGCs had an additive, but not a synergistic effect on promoter activity, strongly suggesting that HIF1A is not co-activated by PGC-1α (Supplementary Material Fig. S3). To interrogate a possible role of the GT repeat size in the activation by HIF1A, we performed co-transfections of SH-SY5Y cells with truncated promoters (− 539 to + 63) harboring various GT repeat sizes and HIF1A expression plasmids (Fig. 4d). Activation of CNS-specific promoters by HIF1A increased up to 21 GTs and a significant interaction was observed between HIF1A and GT size (*p* < 0.0001). The truncated promoter constructs were activated by HIF1A to a similar extent as the 2.0 kbp promoter (matched for GT size). Based on the consensus sequence ((A/G)CGTG) [57], the truncated promoters contained eight putative HREs. Truncation of the CNS promoter to 147 bp (− 84 to + 63) resulted in loss of activation by HIF1A, suggesting that the deleted sequence harbored functional HREs (Fig. 3c). To substantiate this result, we determined the region of the CNS promoter that interacts with HIF1A. We performed ChIP assays using NT2/D1 cells treated with 15 μM CPX or vehicle for 24 h and a monoclonal antibody directed against HIF1A. The region encompassing HREs 5 to 8 and containing 17 GT repeats was readily amplified from NT2/D1 cells treated with CPX, while the same DNA region from NT2/D1 cells without prior CPX treatment was barely amplified. Targeting the proximal promoter region harboring putative HREs 1 to 4 did not produce amplification products in control or CPX treated cells (Fig. 4a–d). A bona fide HRE site [42] of the human *NTRK2* (*TRKB*) promoter used as a positive control was also amplified in CPX treated cells, while no amplification products were noted with the antibody from the exon B5 region serving as negative control. Hence, these results are consistent with the transactivation assays and support promoter activation by HIF1A via HREs located between − 218 and − 84 of the CNS promoter.

**Fig. 5:**
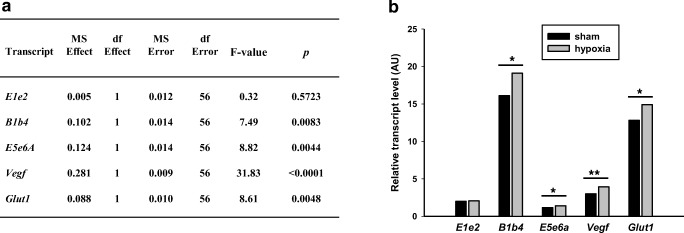
Effect of transient ischemia on the expression of selected transcripts in brains of rats in whom transient forebrain global ischemia of 5 min followed by 1 or 3 h of reperfusion was induced. Samples were collected from 7 brain regions. Sham-operated animals served as controls. Log-transformed transcript levels were used as dependent variables using a 3-way ANOVA with transient ischemia/sham operated, post-perfusion time (1/3 h), and brain regions as independent variables. Note significant effects of transient ischemia for all transcripts analyzed except for *E1e2* which reflects the reference gene transcript; MS mean square; df degrees of freedom (**a**). Average values for all transcripts except for *E1e2* increased in the experimental animals (**b**). *p < 0.01; **p < 0.001

Even though human cell lines were used for the hypoxia experiments, the results may not necessarily emulate the in vivo situation in mammals. Hence, we performed an experiment in rats, whose CNS promoter is conserved and also contains a conserved HRE. Furthermore, we previously described the presence of *B1b4* containing *Ppargc1a* transcripts in mice and rats [16, 31], but *B5e2* transcripts were not detected and such transcripts would have a very short open reading frame of 11 amino acids. To determine whether hypoxia and/or nutrient deprivation influences the expression of CNS-specific *PPARGC1A* transcripts in vivo, we used an established rat model of transient forebrain global ischemia. This model involves the occlusion of both vertebral and carotid arteries and affects all brain regions to a variable degree. To keep the number of animals as low as possible, we used a study design that included transient ischemia/sham, 7 different brain regions, and post-ischemic reperfusion time as independent variables. Transient ischemia resulted in an increase of *Vegf* and *Glut1* transcripts as expected. Furthermore, transcript levels of *B1b4* and *E5e6a*, but not *E1e2*, were also increased (Fig. 5a, b). Thus, the response of CNS-specific transcripts to ischemia occurred in both the rat ischemia model and the human cell lines studied. For all the transcripts measured, effects of brain regions were highly significant, while reperfusion time only affected *Glut1* transcript abundance (Supplementary Material Figs. S4 and S5).

## Discussion

Alternative promoter usage and/or splicing occur in the great majority of mammalian genes and are key mechanisms in transcriptional regulation and generation of protein diversity [58–60]. Several tissue-selective promoters have been described for *PPARGC1A,* but the CNS-specific promoter is unique in that it is localized far upstream of the reference gene promoter and is responsible for the majority of *PPARGC1A* transcripts in human brain, especially in neuronal cells. To this end, we compared some relevant regulations of the CNS-specific and the RG promoter and report not only similar but also distinct factors controlling their activities.

Transcriptional activity of the CNS promoter was modulated by a variable GT tandem repeat region in that a larger GT size enhanced promoter activity. As such, a result was obtained in three neuronal cell types; it is likely to occur also in vivo*.* Hence, the GT size is a likely source of inter-individual variability in human CNS transcript expression. This conclusion is in keeping with a recent study that highlighted the importance of short tandem repeats in the genetic architecture of quantitative human traits. In a survey, more than 2000 tandem repeats were identified that influenced transcript expression. Such repeats were enriched in conserved regions, co-localized with regulatory elements and modulated certain histone marks [61]. In addition, the instable GT microsatellite is part of a highly predicted Z-DNA region. Sequences transiently forming Z-DNAs are non-randomly distributed, occur more frequently proximal to transcription start sites, and influence promoter activity [46–48].

Self- and cross-co-activation of both the RG and CNS promoters by the reference protein and CNS-specific isoforms were consistent findings in our transient transfection studies of SH-SY5Y cells. Even though we did not further identify the transcription factors co-activated in the initial experiments, a synergistic co-activation of ESRRA on both the RG and CNS promoters was clearly demonstrated. Expression profiles of *ESRRA* and *PPARGC1A* transcripts are similar in various tissues and both genes are induced by cold-exposure [62, 63]. An autoregulatory feedback loop consisting of ESRRA response elements in the *ESRRA* and the reference *PPARGC1A* promoters has been discovered previously. The evolutionary conserved ESRRA binding site in the human RG promoter is located 1978-bp upstream of the transcription start site [64], while a 23-bp sequence that contains a functional ERR response element and is present in 1 to 4 copies has been mapped within − 700 bp of the transcription start site of the *ESRRA* promoter [65]. In embryonic fibroblasts of Esrra null mice, the transcriptional activity of Pgc-1α was reduced in comparison to cells from wild-type mice but could be restored by the ectopic expression of Esrra. [66]. We also identified two putative ESRRA binding sites in the CNS promoter, suggesting that a similar feedback loop augments the expression of CNS transcripts. This regulatory loop may be of clinical relevance, as loss of ESRRA function in brain regions of mice or loss-of-function mutations in humans have been associated with eating disorders [67, 68].

Unlike ESRRA, FOXA2 selectively activated the CNS-specific promoter in a positive feedback loop with FL-PGC1α. This result is supported by data showing that adenoviral overexpression of murine Foxa2 in Neuro-2a cells resulted in a dramatic increase in CNS-specific transcripts. In addition, murine Foxa1, another member of the forkhead family of winged-helix transcription factors, induced the transcriptional activity of the CNS-specific promoter in HEK293T cells [52]. FOXA1 and FOXA2 share over 95% homology between mice and humans and near perfect sequence homology in their DNA binding and transactivation domains [69, 70]. The two transcription factors play important, but redundant roles in the development and maintenance of dopaminergic midbrain neurons [51, 71], arguing for the involvement of CNS-specific PGC-1α isoforms. Interestingly, genetic studies implicated the CNS-specific *PPARGC1A* region in the pathogenesis of Parkinson’s disease [31].

PGC-1α, specifically the truncated NT-isoform, has been shown to be upregulated in myotubes by hypoxia, but the canonical hypoxia response pathway characterized by HIF-1α stabilization was not involved [72, 73]. The increase in NT-PGC strongly induced Vegf expression by co-activation of Esrra via conserved binding sites in the *Vegf* promoter and in the first intron. The increased levels of NT-PGC-1α likely result from enhanced alternative splicing and perhaps increased transcription, but the exact mechanisms have not been elucidated. Our results are consistent with these findings as hypoxia, but not HIF1A stabilization, increased the levels of *E5E6A* transcripts. Furthermore, the *E5E6A* level was also increased in the transient ischemia rat model. As demonstrated in the current and previous studies [72, 73], HIF1A does not activate transcription from the reference promoter, but the autoregulatory loop with ESSRA may play a role, as treatment of cardiomyocytes with an ESRRA inverse agonist caused knockdown of Esrra protein expression and blocked the increase in *Ppargc1a* mRNA levels in response to hypoxia [66]. Importantly, the activation of CNS-specific transcripts in hypoxia does occur via the canonical HIF-1α pathway. This conclusion is based on effects of both hypoxia and non-hypoxic HIF1A stabilizing stimuli on CNS-specific *PPARGC1A* transcripts, the activation of CNS-specific promoter reporter constructs by HIF1A expression plasmids, and the data from ChIP assays. In addition, the in vivo results in an established transient ischemia rat model also are consistent with this conclusion. Our data also showed that the average values of *Vegf* and CNS-specific, but not RG transcripts increased after ischemia in all brain regions sampled, except in the cerebellum, where the levels did not change (data not shown). Due to the sample number, the data on individual brain regions must be viewed as preliminary. Clearly, the effects of ischemia require further studies on the time course of response and effects in specific brain regions, as their vulnerability and response may differ. A link between hypoxia and CNS-specific *PPARGC1A* expression is supported by studies in the subterranean rodent *Nannospalax galili*. In this blind mole that survives at 3% O_2_ for up to 11 h as compared to 2–4 h for rats [74], CNS-specific transcripts are upregulated [75].

HIFs interact with HREs with the core sequence 5′-RCGTG-3′ [57], but only a small proportion of the potential binding sites are bound by HIFs across the genome [76]. Several sequence preferences outside the core binding motif have been described but are not absolute. In addition, two or more HREs and/or binding sites for other transcription factors are often found near functional HIF sites and interactions of HIF1A with USF1 and FOXA2 (both located near HREs in the CNS-specific promoter) have been reported [77, 78]. Furthermore, various epigenetic marks are likely playing an important role in HIF binding [76, 79]. An interesting finding of our studies is the interaction of GT repeat size with HIF1A on the transcriptional activity of the CNS promoter. A similar interaction has been described at the NRAMP1 (SLCA11A1) promoter, as an ACGTG site next to a GT microsatellite with high Z-DNA potential affected transcriptional activity and susceptibility to inflammatory and infectious diseases [83]. HIF2A also has been implicated in the hypoxic response, has been shown to trans-activate HRE containing target genes [81], and may therefore influence the expression of the CNS-specific promoter. Indeed, our preliminary studies suggest that HIF2A is expressed in nuclei of CPX-treated cultures and activates the CNS promoter in transient transfection studies (Soyal SM, Patsch W, unpublished results), but additional studies will be required to gain more insight into the regulation of the CNS-specific promoter by HIF2A.

Upregulation of CNS-specific PGC-1α could have major implications for the pathology of cerebral ischemia as well as neuronal physiology. Our results on the interaction of HIF1A and the GT repeat size would imply inter-individual differences in the response to hypoxia. CNS-specific transcripts encode for proteins that differ from the reference protein only at the N-terminus. The N-terminal activation and repression domains appeared to be responsible for gene programs and splicing events that are specific for some PGC-1α isoforms [82]. Our preliminary data in SH-SY5Y in whom the CNS-specific or the RG promoters were selectively upregulated using the CRISPR-dCAS9 approach show overlapping as well as distinct targets for the CNS isoforms and the reference protein. Importantly, CNS-specific forms appear to participate in pathways supporting neural and axonal growth, while the reference protein was mainly related to mitochondrial biogenesis and function (Kwik M, Patsch W, Soyal SM, unpublished results). The generation of new mitochondria, specifically at the peripheral axonal tree, has been shown to be induced by reference PGC-1α and is essential for the axonal growth [83]. Thus, the functions of the CNS isoforms and the reference protein may be intertwined by cross-talk between the CNS and reference proteins as demonstrated by the co-activation of each other’s promoters as shown in the current study.

In conclusion, the CNS-specific, but not the RG, promoter is activated by FOXA2 via an autoregulatory loop, suggesting a role of CNS-specific transcripts in the development and maintenance of dopaminergic mid-brain neurons. The activation of the CNS-specific promoter by HIF1A supports a role of CNS isoforms in ischemia. The hypoxic/ischemic activation may differ between individuals, as an interaction is likely to occur between a variable GT repeat and HIF1A. Finally, cross-talk between the CNS and RG promoters via transcriptional co- and cross-activation may support a balanced expression of the reference protein and the CNS-specific isoforms.

## Electronic Supplementary Material

ESM 1 (DOCX 430 kb)
