# Supplementary material for: The Expression of CNS-Specific PPARGC1A Transcripts Is Regulated by Hypoxia and a Variable GT Repeat Polymorphism
Source: Mol Neurobiol. 2019 Aug 30;57(2):752–64. doi: 10.1007/s12035-019-01731-5 (PMC7031416; doi:10.1007/s12035-019-01731-5)
Supplement: Supplementary file 1 — (DOCX 430 kb) [file 12035_2019_1731_MOESM1_ESM.docx]

**Supplementary Table S1.**

**Amplification Primers for cloning CNS-PPARGC1A promoters with various GT expansions and promoters truncated to 147bp into pGL4.11**

| CNS-specific 602 bp *PPARGC1A* prom (including 11GT microsatellite region) *KpnI* forward | 5´ GAGA*ggtacc*CAGGAGGTTGCCGGACGTAG 3´ |
| --- | --- |
| CNS-specific 147 bp *PPARGC1A* prom *KpnI* forward | 5´ GATATA*ggtacc*GCTCGCTCATTATCTCGCCCTC 3´ |
| CNS-specific *PPARGC1A* prom *HindIII* reverse | 5´ GAGA*aagctt*ATAGGTTCGTCCTGACTTGGGC 3´ |

**RT/PCR Amplification primers for human transcripts**

| *PPARGC1A* Exon B1 forward | 5´ TACAACTACGGCTCCTCCTGG 3´ |
| --- | --- |
| *PPARGC1A* Exon B4 reverse | 5´ TACCCTTCATCCATGGGGCTC 3´ |
| *PPARGC1A* Exon B5 forward | 5´ CCTGGCTGCTGCTTTGGTA 3´ |
| *PPARGC1A* Exon 1 forward | 5´ TGAGTCTGTATGGAGTGACATCGAGTG 3´ |
| *PPARGC1A* Exon 2 reverse | 5´ GCTGTCTGTATCCAAGTCGT 3´ |
| *PPARGC1A* Exon 5 forward | 5´ TCACACCAAACCCACAGAGAAC 3´ |
| *PPARGC1A* Exon 6A reverse | 5´ GGTCACTGGAAGATATGGCACATT 3´ |
| *VEGF* Exon 3 forward | 5´ GGAGTACCCTGATGAGATCGAG 3´ |
| *VEGF* Exon 4 reverse | 5´ CTCATCTCTCCTATGTGCTGGC 3´ |
| *RPLP0* forward | 5´ GGCACCATTGAAATCCTGAGTGAT 3´ |
| *RPLP0* reverse | 5´ TTGCGGACACCCTCCAGGAAGC 3´ |

**RT/PCR Amplification primers for rat transcripts**

| *Ppargc1a* Exon B1 forward | 5´ TACAACTACGGCTCCTCCTGG 3´ |
| --- | --- |
| *Ppargc1a* Exon B4 reverse | 5´ TACCCTTCATCCATGGGGCTC 3´ |
| *Ppargc1a* Exon 1 forward | 5´ CTTGGGACATGTGCAGCCAAG 3´ |
| *Ppargc1a* Exon 2 reverse | 5´ GCTGTCTGTGTCCAGGTCAT 3´ |
| *Ppargc1a* Exon 5 forward | 5´ CACAACCGCAGTCGCAACAT 3´ |
| *Ppargc1a* Exon 6A reverse | 5´ GGTCACTGGAAGATATGGCACATT 3´ |
| *Vegf* Exon 3 forward | 5´ GAGACCCTGGTGGACATCTTC 3´ |
| *Vegf* Exon 4 reverse | 5´ CTCATCTCTCCTATGTGCTGGC 3´ |
| *Glut1* Exon 9 forward | 5´ GACCTGCTGCTGTTGCTGTG 3´ |
| *Glut1* Exon 10 reverse | 5´ GATGAAGAAGAGTACCAGCACCG 3´ |
| *Rplp0* forward | 5´ GGTACCATTGAAATCCTGAGCGAT 3´ |
| *Rplp0* reverse | 5´ TTGCGGACACCCTCTAGGAAGC 3´ |

**Amplification primers for CHIP**

| *PPARGC1A* CNS promoter Probe 1 forward | 5´ CCAAACAGACGCACACCCAG 3´ |
| --- | --- |
| *PPARGC1A* CNS promoter Probe 1 reverse | 5´ AGAGGGCGAGATAATGAGCGA 3´ |
| *PPARGC1A* CNS promoter Probe 2 forward | 5´ CATTATCTCGCCCTCTCGCTTC 3´ |
| *PPARGC1A* CNS promoter Probe 2 reverse | 5´ GGTTCGTCCTGACTTGGGCA 3´ |
| *PPARGC1A* Exon B5 (Negative Control) forward | 5´ CCTGGCTGCTGCTTTGGTA 3´ |
| *PPARGC1A* Exon B5 (Negative Control) reverse | 5´ GCCTGCTTCTCGCTGAAGTA 3´ |
| *TrkB* (NTRK2) promoter forward (Positive Control) | 5´ GAAGCAGACAGCAGCAGCATGTG 3´ |
| *TrkB* (NTRK2) promoter reverse (Positive Control) | 5´ CGCGATTGTAGAAGAGACTGTGGT 3´ |

**Fig. S1** Effect of a common GT-repeat polymorphism on CNS-specific promoter activity in NT2/D1 cells (left) and HT22 cells (right). Proximal CNS-promoters from human subjects harboring 11 GT- or 21 GT-repeats, but otherwise of identical sequence (-539 to + 63 bp relative to the transcription start site in the common allele with 11GTs) were cloned in reporter vectors and transiently transfected into NT2/D1 cells (left) or HT22 cells; *, *p*<0.001

**Fig. S2** The iron chelator deferoxamine (DFO) increases the cellular levels of *VEGF* and the CNS-specific transcripts *B1B4*, but not the reference gene transcripts *E1E2*. SH-SY5Y cells were cultured without or with 20 µM DFO for 1 or 3 days; *, *p*<0.01; **, *p*<0.001

**Fig. S3** The CNS-specific promoter is activated by HIF1A, but HIF1A activation is not co-activated by PGC-1α. Reporter constructs containing the CNS-promoter (2 kbp, 17 GT repeats) were transiently transfected into SH-SY5Y cells along with expression plasmids encoding HIF1A, PGC-1α or both. Activities were analyzed by two-way ANOVA. Effects of HIF1A, PGC1α or both vs. promoter without expression plasmids all <0.001; no significant interaction between HIF1A and PGC-1α was noted (p=0.414) and the sum of activations by individual transfections of HIF1A or PGC-1α did not differ from the activation obtained by co-transfections of HIF1A and PGC-1α (p=0.481) strongly suggesting an additive effect. Similar effects of transfections with expression plasmids encoding FL-PGC-1α or FL-B4-PGC1α were observed; *, *p*<0.001

**Fig. S4** Associations of brain regions with the expression of selected transcripts in rats in whom transient forebrain global ischemia of 5 min followed by 1 or 3 h of reperfusion was induced. Samples were collected from 7 brain regions; FC, frontal cortex; STR1, dorsolateral part of striatum; STR2, rest of striatum; HCA1, hippocampus CA1 region; HDG, hippocampus dentate gyrus and CA3 region; THAL, thalamus; CER, cerebellum. Log-transformed transcript levels were used as dependent variables using a 3-way ANOVA with brain regions, post-perfusion time (1/3 h) and ischemia/sham operated as independent variables. MS, mean square; df, degrees of freedom; all transcripts measured differed significantly by regions (A). Average values (SE) of transcript levels for all regions (B-G)

**Fig. S5** Effect of post-perfusion times on the expression of selected transcripts in rats in whom transient forebrain global ischemia of 5 min followed by 1 or 3 h of reperfusion was induced. Samples were collected from 7 brain regions. Log-transformed transcript levels were used as dependent variables using a 3-way ANOVA with post-perfusion time (1 h vs. 3 h), brain regions and ischemia/sham operated as independent variables. MS, mean square; df, degrees of freedom; GLUT1 transcript levels differed by post-perfusion time (A). Average transcript levels by perfusion time (B). *, p<0.001
